# The Weight of Cognitions in Panic: The Link between Misinterpretations and Panic Attacks

**DOI:** 10.1371/journal.pone.0070315

**Published:** 2013-08-05

**Authors:** Klara De Cort, Dirk Hermans, Daphne Noortman, Wiesje Arends, Eric J. L. Griez, Koen R. J. Schruers

**Affiliations:** 1 Maastricht University, Institute for Mental Health and Neuroscience, Maastricht, The Netherlands; 2 University of Leuven, Center for Learning and Experimental Psychopathology, Leuven, Belgium; The University of Queensland, Australia

## Abstract

In cognitive theory it is hypothesized that panic attacks are provoked by catastrophic misinterpretations of bodily sensations. The aim of the present study was to investigate the ability of associated word pairs referring to catastrophic thinking (e.g. palpitations-heart attack) in producing panic attacks. Patients with PD (n = 20), patients with mixed anxiety disorders (n = 20), and a healthy control group (n = 30) participated in the present study. To enhance ecological validity we first conducted a stimulus validation experiment. Subsequently, nine suitable panic and neutral word pairs were presented in block to the participants. Anxiety levels were assessed before and after the presentation. PD patients were more anxious when reading these word pairs, compared to neutral word pairs. However, none of the participants experienced a panic attack upon reading the word pairs. From the present results it seems that catastrophic thinking is rather related to the anticipatory anxiety for panic attacks, but not necessarily with the occurrence of the panic attacks themselves.

## Introduction

Persons suffering from panic attacks often believe that they are having an acute medical condition and might die at any moment. Cognitive theories [1]–[5] of Panic Disorder (PD) state that these beliefs play a causal role and are therefore crucial in the pathogenesis of panic attacks. Typical beliefs that are held by PD patients are viewed as catastrophic misinterpretations of physical sensations. Specifically, interoceptive stimuli are erroneously interpreted as evidence of an impending disaster, related to their well-being. This misinterpretation results in anxiety and increased bodily sensations, thereby reaffirming the initial misinterpretation. As a consequence, PD patients become hyper vigilant for bodily sensations resulting in an ‘attentional bias’ towards physical cues associated with panic. As a result, the patient is more likely to re-activate the circle of misinterpretation.

The core hypotheses of this cognitive perspective can be summarized as follows:

1. Bias specificity: PD patients have specific catastrophic misinterpretations concerning their physical sensations.
2. Disorder specificity: This interpretative bias is assumed to be only specific for PD patients (in contrast with patients suffering from other anxiety disorders).
3. Causality: These specific catastrophic cognitions are regarded as sufficient and necessary in the production of panic attacks.

There is evidence in support of cognitive misinterpretation in PD patients. This includes the use of retrospective [6] and prospective self-reports [7], [8], priming tasks [4] and questionnaires [9]–[14]. The evidence that these biases are particular and specifically apply to PD patients is less well documented. Much of the conducted studies are based on the Interpretation Questionnaire of McNally and Foa [9]. In this study PD patients and normal controls were asked to read ambiguous scenarios describing either a bodily sensation (e.g. “You feel discomfort in your chest area. Why?”) or an external event (e.g. “Your phone rings at 3 AM. Why is someone calling at this time?”). PD patients were significantly more likely than normals to interpret both scenarios in a negative fashion, but only when a broad criterion of harm was used (e.g. “I’m going to panic” instead of the narrow criterion of harm; “I’m having a heart attack”). These non-specific results have been replicated by Harvey et al. [10]. PD patients were significantly more likely to choose a broad negative interpretation for the bodily sensation, and did not differ from patients suffering from social phobia on the interpretation of the external event. Austin and Richards [13], in replicating Clark et al. [11], were the first to demonstrate that PD patients made more specific anxiety harm-related interpretations (e.g. I’m having a heart attack, narrow criteria of harm) than anxious and non-anxious controls. However, the study failed to find a difference between PD patients and social anxiety patients, with regard both to internal and external stimuli. An internet study of Austin and Kiropoulos [14] was the first to find a specific catastrophic interpretation bias in PD patients in contrast to social anxiety and non-anxious controls. Further, factor analytic and correlational analyses revealed that specific somatic sensations in PD are related to specific catastrophic thoughts [4], [12]. However, no distinctions were made between PD patients and others.

Despite this rather large amount of studies, the pattern of findings only partially give support to the first two hypotheses concerning bias and disorder specificity.

The last premise of the cognitive perspective concerns the hypothesized causal role of specific catastrophic cognitions. It is hypothesized that catastrophic cognitions of bodily sensations are sufficient and necessary in evoking panic attacks in PD patients. However, to date, this has never been directly tested. There are some experimental studies which give an indication of more catastrophic misinterpretations during experimentally induced panic attacks in patients suffering from PD in contrast with controls [15], [16]. As Clark [17] (p 75) stated “the cognitive theory of panic which proposes that the challenge tests induced panic because they produce sensations that panic patients are prone to misinterpret and that it is the misinterpretation which is responsible for the induced attack”. However, in those studies panic attacks were induced in the laboratory, by CO_2_ inhalations or hyperventilation, not (only) through catastrophic thinking, so it is difficult to determine the actual cause of the panic attack. Further, catastrophic thinking was only assessed after the manipulation. There is growing evidence in support for the efficacy of cognitive therapy in the treatment of PD [18], [19]. This success of cognitive therapy is often cited as support in favor of the causal role of cognitive misinterpretations in PD [18], [19]. Recent research is studying the mediating role of cognitive change during treatment [20], [21]. Cognitive theory states that improvement during treatment is mediated by changes in catastrophic thinking. Teachman et al. [21] demonstrated with the Implicit Association Test [22], a measure of automatic panic associations, that over the course of cognitive behavioural treatment automatic panic associations changed and that these changes were correlated with symptom improvement. However, the possibility that cognitive change is simply an epiphenomenon of panic, and not meaningfully related to symptom reduction needed to be addressed further. In a next study of Teachman, Marker & Clerkin [23] was demonstrated that the slope of change in misinterpretations over the course of cognitive behavioural therapy acted as a predictor of reductions in panic symptoms. On the basis of these results the authors suggested that cognitive biases are functionally related and therefore not an epiphenomenon or consequence of panic. However, they also agree that their design does not test causality. Furthermore, it was found that breathing training focused on changing pCO_2_ and cognitive therapy focused on manipulating maladaptive cognitions led both to reductions in fears of bodily sensations and symptom appraisal. However, changing fears of bodily sensations or changing cognitions did not predict changes in respiration [24]. Accordingly, the fact that cognitive therapy is successful does not automatically imply that faulty cognitions are the cause of panic.

There is, however, one report of an uncontrolled experiment by Clark et al. [4] where only associated word pairs (e.g; palpitations-dying, dizziness-fainting; Paired Associated Task) where used to activate catastrophic thinking. As cited by Clark (p152) [4] as a prediction following from the causality hypothesis “within panic patients conditions which activate catastrophic misinterpretations should lead to an increase in anxiety and panic”. It was stated that 83% of PD patients experienced a panic attack while reading word pairs, in contrast with healthy subjects and recovered PD patients. A panic attack was defined as a sudden increase in anxiety reaching at least 50 on a 100-point scale and accompanied by four or more symptoms. While cited in several books and papers [25], [26] and despite the obvious heuristic value, this observation was never formally replicated in a controlled experiment.

In sum, while there is evidence for catastrophic misinterpretation in PD patients, there is only equivocal support for bias and disorder specificity. Further, because of the lack of well controlled studies, the available evidence cannot entirely account for the proposed causal role of cognitions in the cognitive theory of panic attacks [27], [28]. To date, support for the three proposed hypotheses is rather limited.

The aim of the present study is to experimentally test the last hypotheses concerning the causal role of catastrophic misinterpretations of physical sensations in eliciting panic attacks in PD. We wanted to experimentally replicate the report of Clark et al. [4], in attempting to provoke panic attacks by presenting series of associated word pairs referring to catastrophic interpretation (e.g. palpitations-dying). The present study consists of two experiments. To enhance the validity of our study we first conducted a stimulus validation experiment. We wanted to select anxiety provoking stimuli to use in our controlled experiment. After selecting our word pairs, they were presented in block and anxiety levels were assessed. We hypothesized that only PD patients would display a panic reaction upon reading a block of panic word pairs compared with controls and in contrast with neutral word pairs. A panic attack was defined as an increase in anxiety reaching at least 50 on a 100-point scale. Further, it was expected that a block of panic word pairs would produce more anxiety than a block of neutral word pairs.

## Experiment 1

Experiment 1 was designed to find suitable word pairs to use in our second Experiment.

### Materials and Methods

The study was approved by the Maastricht University Medical Ethics Committee.

#### Participants

This experiment involved three groups of participants. In addition to the panic group, an anxious control group and a non-clinical control group were included in the design. Patients were recruited while seeking treatment at the Academic Anxiety Center in Maastricht. Healthy volunteers were recruited via advertisement. Inclusion criteria for all participants involved age between 18–70 years and good physical health. Exclusion criteria were the presence of dyslexia and a psychotic or dissociative disorder on axis I of DSM-IV criteria.

In the panic group, 21 patients (12 female) who met the DSM-IV criteria for PD were selected. Mean age was 42 years (*SD* = 12.6). Thirty-three percent had co-morbid depression.

The ‘anxious control’ group consisted of 20 patients (14 female). Of these, 35% had obsessive compulsive disorder, 25% social phobia, 40% specific phobia as their primary diagnosis. Their mean age was 36 years (*SD* = 13.9). Twenty five percent had co-morbid depression and two participants had co-morbid specific phobia.

The ‘healthy control’ group consisted of 30 participants (12 female). This group was matched with the panic group for sex and age. Participants had never had a psychiatric disorder. Mean age was 43 years (*SD* = 11.3). They were recruited by advertisement.

#### Questionnaires

The presence of possible mental disorders was evaluated by a trained clinician by means of the *Mini International Neuropsychiatric Interview* [29], based on DSM-IV criteria. All patients were also administered the *Panic Agoraphobia Scale* (PAS) [30] and the *State Trait Anxiety Inventory-trait* version (STAI-T) [31]. The PAS is a 13– item clinical interview assessing panic attacks, anticipatory anxiety and avoidance behavior, with a range between zero and 52. Previous work has demonstrated that the PAS has satisfactory values for internal consistency, test-retest reliability and correlations with other anxiety scales. The STAI-T is a self-rating scale to asses general anxiety and has good validity and reliability (a >.90) [32].

Anxiety levels were measured with an *electronic Visual Analogue Scale* (eVAS). The eVAS was programmed on a Compaq Tablet PC, TC1000, with a 21,0 cm × 16,0 cm touch screen having a 1027 × 748 pixel resolution. The eVAS was a 20 cm × 1 cm horizontal bar. Participants had to mark their anxiety level by tipping on the bar with a stylus, which had a 1 mm diameter spherical tip. The scale was anchored from 0, “no anxiety at all”, to 100, “the worst imaginable anxiety”. This instrument has been validated for use during 35% CO_2_ challenges [33]. Additionally, *The Panic Symptom List* (PSL) was used to measure panic symptoms. This list consists of 13 symptoms that can occur during a panic attack. Participants had to score the occurrence of bodily sensations on a five-point Likert scale, ranging from “not at all” to “very much”. To assess mood state, the short version of the *Profile of Mood States* (POMS) [34] was used. It consists of 32 items referring to five mood states; depression, anger, fatigue, tension and will-power. Participants had to score their current mood states on a five-point Likert scale from “not at all” to “very much”. Depressive symptoms were assessed with the *Montgomery-Asberg Depression Rating Scale* (MADRS) [35] and the *Self-rating Depression Scale* (SDS) [36]. The MADRS is a ten item clinical rating scale to assess severity of depressive symptoms. Previous research showed that the MADRS is characterised by excellent internal consistency [37]. We used the Dutch version [38]. The SDS is a self-report measure of depression consisting of 20 items, with a four-point scale ranging from ‘a little of the time’ (1) to ‘most of the time’ (4). Research supports the validity of this measure [39].

#### Paired Associated Task

In the Paired Associated Task, two categories of words were used: 10 panic-related words and 10 neutral words. Eight threat words were derived from Clark et al. [4] and consisted of a symptom and its consequence. To make a complete set of 10 words we added two words, relying on former research [12]. The neutral words were neither positive nor abstract. To rule out a possible difference in results by presentation form, all categories were matched for word length and imageability. Stimuli were presented on a personal computer. The words appeared in 12-cm block letters on a Philips colour monitor.

#### Procedure

In the pre-test screening, all questionnaires were administered after obtaining written informed consent.

In the test phase, to measure possible experimental anticipatory anxiety, participants were asked to score their anxiety level on the eVAS. After the procedure and the aim of the experiment was explained, the participants were asked to fill in the eVAS, PSL and POMS as pre-measurement.

The task was presented on a computer screen. Participants were given the instruction to read the word-pairs in silence. Word-pairs were presented separately with an inter-trial interval that ranged between 12 and 20 seconds. Presentation time was 8 seconds. The presentation order of the word pairs was randomized. After each word pair, participants were asked to score their anxiety level on the eVAS. At the end of the experiment, participants were asked again to score their anxiety level, to fill in the PSL and the POMS.

#### Design

The eVAS, PSL and POMS scores were used as main outcome variables. The Kolmogorov-Smirnov test was used to test the normality of these variables. Not all variables were normally distributed. Hence, all analyses were performed with non-parametric tests. Therefore median ( = *M*) values are presented in the text. First the Friedman and the Kruskal-Wallis Test were performed to explore for significant differences in the types of word-pairs used and/or in the different groups. If significant differences were noted, the Mann-Whitney Test was performed for the analyses of differences between the different groups and the Wilcoxon Test for the analyses between the different types of word associates. The separate word pairs were analyzed with the Kruskal-Wallis Test. Differences between the groups for eVAS, PSL and POMS assessed during the experiment were analyzed with the Mann-Whitney Test. Differences within the groups were analyzed with the Friedman and Wilcoxon Test.

Non-parametric Spearman correlations were used to analyze the relationship between depressive symptoms and anxiety levels.

Statistical significance for all analyses was accepted at a significance level of *p*<.05 (2-tailed).

### Results

#### Questionnaires

As expected, the panic group had significantly higher scores on the PAS, as compared to the anxious control group, *t*(39) = 4.30, *p*<.001. The two groups did not differ in their level of depression, as indicated by the scores on the SDS, *t*(39) = 1.251, *n.s.*, and the MADRS, *t*(39) = 0.038, *n.s.* Similarly, both patient groups did not differ on trait anxiety, *t*(55) = 1.4, n.s. See Table 1 for the clinical and demographic characteristics of experiment 1.

**Table 1:** Mean values for age, gender, MADRS, SDS, PAS and STAI-T for panic group (PG), anxious controls (AC) and healthy controls (HC) in Experiment 1.

	gender	Age	MADRS	SDS	PAS	STAI-T
**PG**	0.57	42 (12.6)	12.7 (7.4)	47 (8)	24 (10.4)*	53 (10.4)
**AC**	0.7**	36 (13.9)	12.6 (11.4)	43.1 (12)	10 (10.2)*	48.7 (14)
**HC**	0.4	43 (11.3)				

Standard deviations are presented in brackets.

Note: MADRS = Montgomery-Asberg Depression Scale; SDS = Self-rating Depression Scale; PAS = Panic and Agoraphobia Scale; STAI-T = State Trait Anxiety Inventory-Trait *significant differences between the groups and between experiment 1&2 **; *p*<.05.

While entering the experiment, panic patients were significantly more anxious than the anxious control patients and both patient groups were significantly more anxious than the healthy volunteers, χ^2^(3, N = 71) = 26.36, *p*<.001. At the end of the experiment panic patients were still more anxious and reported more symptoms than the other groups (see Table 2). Further, anxious control patients were also more anxious and reported more symptoms than healthy volunteers, eVAS before: χ^2^(3, N = 71) = 29.44, *p*<.001, eVAS after: χ^2^(3, N = 71) = 30.13, *p*<.001, PSL before: χ^2^(3, N = 71) = 33, *p*<.001, PSL after χ^2^(3, N = 71) = 29.3, *p*<.001. Mood state was similar for the two patient groups, but was significant lower than the healthy volunteers; POMS before: *U* = 53, N_1_ = 20, N_2_ = 30, *p*<.001; *U* = 64, N_1_ = 21, N_2_ = 30, *p*<.001, POMS after: *U* = 66, N_1_ = 20, N_2_ = 30, *p*<.001; *U* = 51, N_1_ = 21 N_2_ = 30, *p*<.001. Within all groups were no differences in assessment for eVAS and PSL measurements. So, during the experiment anxiety levels and symptom reporting stayed the same for all participants (*n.s.*). For POMS, however, significant differences were noted within the healthy volunteers. Healthy volunteers had a significantly better mood state after the experiment than before; *z* = −2.5, N-Ties = 27, *p*<.012. Within the patient groups were no significant differences concerning mood state (*n.s.*).

**Table 2:** Median values for (e)VAS, PSL and POM(S) for panic group (PG), anxious controls (AC) and healthy controls (HC) in Experiment 1.

	eVASentr	Pre-eVAS	Pos-eVAS	Pre-PSL	Pos-PSL	Pre-POM	Pos-POM
**PG**	19	22	35	6	6	32	38
**AC**	6	5	4	1	1	32	27
**HC**	0	0	0	0	0	7	5

eVAS ent = eVAS entrance, Pre = before start experiment, Pos = at the end of experiment.

#### Paired associated task

All participants were significantly more anxious when reading panic-relevant word pairs in contrast with neutral word pairs, χ^2^(1, N = 71) = 45.56, *p*<.001, *M*_panic_ = 12.1, *M*_neutral = _1.2. Further, there were significant group differences both for panic, χ^2^(2, N = 71) = 24.35, *p*<.001 and for neutral, χ^2^(2, N = 71) = 21.94, *p*<.001, word pairs.

As expected, panic patients were significantly more anxious with respect to panic word pairs compared to anxious control patients, *U* = 96.5, N_1_ = 21, N_2_ = 20, *p*<.03, *M*_panic_ = 51.8, *M*_anxious controls_ = 18.7 and healthy volunteers, *U* = 78, N_1_ = 21, N_2_ = 20, *p*<.001, *M*_healty volunteers_ = 1.5. There were also significant differences between anxious control patients and healthy volunteers both for panic word pairs, *U* = 169.5, N_1_ = 20, N_2_ = 30, *p*<.01, *M*_anxious controls = _18.7, *M*_healty volunteers_ = 1.5 and for neutral word pairs, *U* = 133.5, N_1_ = 20, N_2_ = 30, *p*<.001, *M*_anxious controls = _2.65, *M*_healty volunteers_ = 0.6. Furthermore, there were significant differences between panic patients and healthy volunteers concerning neutral word pairs, *U* = 91, N_1_ = 21, N_2_ = 30, *p*<.001, *M*_panic_ = 5.2, *M*_healty volunteers_ = 0.6. There were, however, with respect to neutral word pairs no significant differences between the two patient groups *U* = 162, N_1_ = 21, N_2_ = 20, *n.s.*, *M*_panic_ = 5.2, *M*_anxious controls = _2.65.

**Figure 1:**
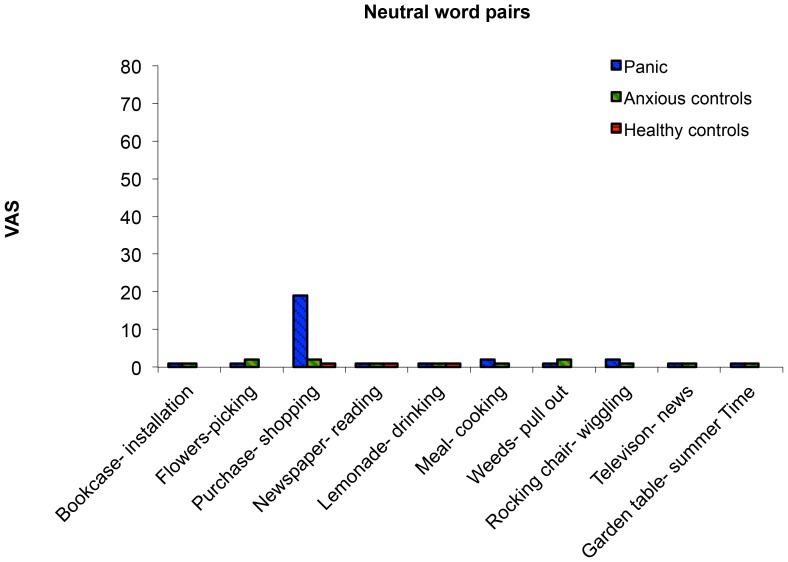
Median level of anxiety on the electronic Visual Analogue Scale. Neutral word pairs for panic group, anxious controls and healthy controls in Experiment 1.

Inspection of the anxiety levels of the neutral word pairs (see Figure 1) demonstrated that all word pairs were close to zero, with the exception of purchase-shopping. Analyses indeed show that for this word pair significant differences between the panic group and healthy controls, *U = *116.5, N_1_ = 21, N_2_ = 30, *p*<.001 were noted. In retrospect, this word pair was probably strongly related to the agoraphobia component in the panic group.

**Figure 2:**
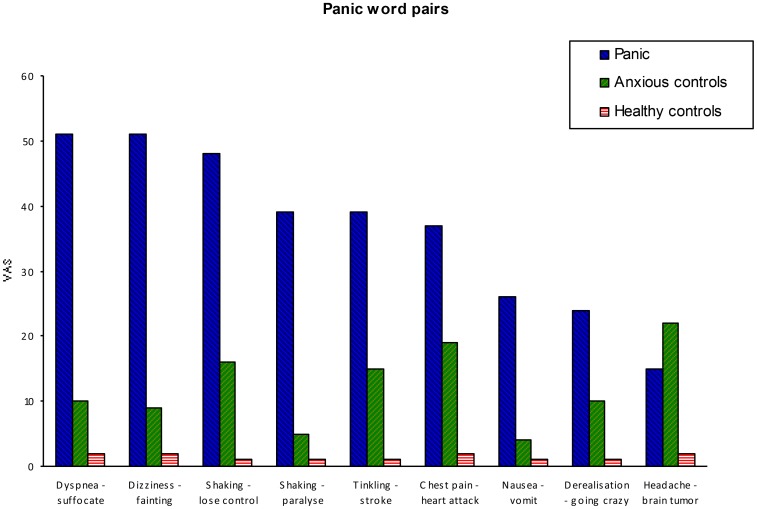
Median level of anxiety on the electronic Visual Analogue Scale. Panic word pairs for panic group, anxious controls and healthy controls in Experiment 2.

In the panic word pairs (see Figure 2) we also noticed one outliner, namely headache-brain tumor. This word pair provokes equal anxiety in the patient groups and in the controls, *U = *210.5, N_1_ = 20, N_2_ = 30, *n.s.*; *U = *178, N_1_ = 21, N_2_ = 20, *n.s.*; *U = *157, N_1_ = 21, N_2_ = 30, *p*<.002. Therefore, also this word pair was not appropriate as panic word pair.

To assess the relationship between depressive symptoms and anxiety levels on the word pairs correlations were calculated between MADRS scores and mean differences scores between panic and neutral word pairs within the patient groups. There were no significant correlations, nor for panic patients or anxious control patients, *r_s_* = −0.03, N = 21, *n.s.*; *r*_s_ = −0.13, N = 20, *n.s.*

## Experiment 2

Experiment 2 was designed to experimentally test the causal role of catastrophic misinterpretations in eliciting panic attacks. This experiment used a sample of participants that was independent of experiment 1.

### Materials and Methods

The study was approved by the Maastricht University Medical Ethics Committee.

#### Participants

The study involved three groups of participants: a panic group, an anxious control group and a non-clinical control group. Patients were recruited while seeking treatment at the Academic Anxiety Center in Maastricht. Healthy volunteers were recruited via advertisement. Inclusion criteria for all participants involved age between 18–70 years and good physical health. Exclusion criteria were the presence of dyslexia and a psychotic or dissociative disorder on axis I of DSM-IV criteria.

In the panic group, 20 patients (11 female) were selected who met the DSM-IV criteria for PD. Mean age was 40.7 years (*SD* = 13.5). Forty-five percent had co-morbid depression.

The ‘anxious control’ group consisted of 20 patients (9 female). Of these, 30% had obsessive compulsive disorder, 25% social phobia, 35% specific phobia, 10% generalized anxiety disorder as their primary diagnosis. Their mean age was 41.3 years (*SD* = 15.1). Twenty-five percent had co-morbid depression, two participants had co-morbid specific phobia, one participant had hypochondriasis.

The ‘healthy control’ group consisted of 30 participants (17 female). This group was matched with the panic group for sex and age. Participants had never had a psychiatric disorder. Mean age was 38.3 years (*SD* = 12.5). They were recruited by advertisement.

#### Questionnaires

The same questionnaires were used as in Experiment 1.

#### Paired associated task

In the Paired Associated Task, two categories of word pairs were used: 9 panic-related and 9 neutral word pairs. These 18 word pairs derived from Experiment 1. Nine panic-related word pairs were considered suitable for Experiment 2. Namely, these word pairs provoked more anxiety in panic patients in contrast with healthy controls and/or other anxious controls. Only ‘headache-brain tumor’ provoked no differences in anxiety between the patient groups and between the control groups. This word associate was removed for Experiment 2. Overall all neutral word pairs were suitable for experiment 2, with the exception of ‘purchase-shopping’. This word associate provoked some anxiety in panic patients and was therefore not appropriate as neutral word. The stimuli were presented in block on a personal computer. The words appeared in 12-cm block letters on a Philips colour monitor.

#### Procedure

In the pre-test screening, all questionnaires were administered after obtaining written informed consent.

In the test phase, when entering the experiment, patients scored their anxiety level on the eVAS. After more information was given about the experiment participants were asked to fill in the PSL, POMS and eVAS. The task was presented on a computer screen. Before each block of word pairs the participants were asked to fill in the eVAS ( = pre-eVAS). Further, participants were given the instruction to read the word-pairs. Word-pairs were presented in block (instead separately) in line with the anecdotecal report of Clark et al. [4]. After each block of word-pairs participants were asked to fill in the eVAS to score their highest anxiety level while reading the word pairs ( = post-eVAS). There were 2 blocks, a panic and a neutral one. Order of blocks was counter-balanced. Presentation time was 3 seconds, with and interval ratio of 2 seconds. The word pairs were randomized. Between each block, participants had to fill in a short questionnaire to prevent possible cross-over effects. At the end of the experiment participants were asked again to score their anxiety level, to fill in the PSL and the POMS.

#### Design

First, the difference between pre- and post-eVAS was calculated for each block of word–pairs ( = delta-eVAS). The median delta-eVAS ( = *M*) for both the panic and neutral block of word-pairs was calculated. Because not all variables were normal distributed all analyses were performed with non-parametric tests. The same tests were used as in Experiment 1.

### Results

#### Questionnaires

The pattern of results for the questionnaires (see Table 3) was very similar to that of Experiment 1. The panic group scored significantly higher on the PAS, as compared to the anxious control group, *t*(38) = 7.22, *p*<.001. Similarly, both patient groups did not differ in their level of depression, as indicated by the scores on the SDS, *t*(38) = 1.89, *n.s.*, and the MADRS, *t*(38) = 1.62, *n.s.* The patient groups did not differ in trait anxiety, t(38) = 1.3, n.s. See Table 1 & 3 for the demographics and clinical characteristics of both samples. Only significant differences for gender between the anxious control groups of the two experiments were noted, t(43) = −2.2, *p*<.033.

**Table 3:** Mean values for age, gender, MADRS, SDS, PAS and STAI-T for panic group (PG), anxious controls (AC) and healthy controls (HC) in Experiment 2.

	gender	Age	MADRS	SDS	PAS	STAI-T
**PG**	0.55	41 (13.5)	15 (7.6)	47 (11.2)	25 (10.3)*	51 (10.7)
**AC**	0.45	41 (15)	10.6 (9.1)	41 (10.6)	4.5 (8)*	46 (15)
**HC**	0.6	38 (12.5)				

Standard deviations are presented in brackets.

Note: MADRS = Montgomery-Asberg Depression Scale; SDS = Self-rating Depression Scale; PAS = Panic and Agoraphobia Scale; STAI-T = State Trait Anxiety Inventory-Trait. *significant differences between the groups and between experiment 1&2 **; *p*<.05.

While entering the experiment all patients were significantly more anxious than the healthy controls, χ^2^(3, N = 69) = 11.21, *p*<.004 (see Table 4). At the end of the experiment all patients stayed anxious, reported more symptoms and had a lower mood state in contrast with healthy volunteers, eVAS before: χ^2^(3, N = 69) = 15.94, *p*<.001, eVAS after: χ^2^(3, N = 69) = 17.91, *p*<.001, PSL before: χ^2^(3, N = 69) = 23.35, *p*<.001, PSL after χ^2^(3, N = 69) = 27.4, *p*<.001, POMS before: χ^2^(3, N = 71) = 21.19, *p*<.001, POMS after: χ^2^(3, N = 69) = 21.8, *p*<.001. There were no significant differences between the patient groups, *n.s.*There were no significant differences within the different groups for eVAS, PSL and POMS, *n.s.*

**Table 4:** Median values for (e)VAS, PSL and POM(S) for panic group (PG), anxious controls (AC) and healthy controls (HC) in Experiment 2.

	eVAS-entr	Pre-eVAS	Pos-eVAS	Pre-PSL	Pos-PSL	Pre-POM	Pos-POM
**PG**	19	23	18	3	1	28	29
**AC**	15	12	13	1	3	27	24
**HC**	1	1	0	0	0	9	9

eVAS ent = eVAS entrance, Pre = before start experiment, Pos = at the end of experiment.

#### Paired associated task

There were significant group differences both for panic, χ^2^ (2, N = 70) = 14.5, *p*<.001 and for neutral, χ^2^ (2, N = 70) = 16, *p*<.001, word pairs. Between the patient groups were no significant differences nor for panic, *U = *167, N_1_ = 20, N_2_ = 20, *n.s.*, *M*_panic group_ = 3.5, *M*_anxious controls_ = 3, or for neutral, *U = *168, N_1_ = 20, N_2_ = 20, *n.s.*, *M*_panic group_ = 3, *M*_anxious controls_ = 1, word pairs. There were, however significant differences between the patients and the healthy controls, for neutral block: *U = *121, N_1_ = 20, N_2_ = 30, *p*<.001; *U = *167, N_1_ = 20, N_2_ = 30, *p*<.004, *M*_panic group_ = 3, *M*_anxious controls_ = 1, *M*_healthy controls_ = 0.1; for panic block: *U* = 120, N_1_ = 20, N_2_ = 30, *p*<.001, *U = *181, N_1_ = 20, N_2_ = 30, *p*<.012, *M*_panic group_ = 3.5, *M*_anxious controls_ = 3, *M*_healthy controls_ = 0.1. Overall, there were no significant differences between the two wordtypes, χ^2^ (1, N = 70) = 3.3, *n.s.*, *M*_panic words_ = 1, *M*_neutral words_ = 1. Within the panic group, however, the panic block of word pairs evoked significantly more anxiety than the neutral block of word pairs, *z = *1.96, N-Ties = 15, *p*<.5, *M*_panic words_ = 3.5, *M*_neutral words_ = 3. There were no differences in anxiety levels between panic and neutral block of word pairs for anxious, *z = *1.16, N-Ties = 12, *n.s.*, *M*_panic words_ = 3, *M*_neutral words_ = 1 and healthy, *z = *1.5, N-Ties = 17, *n.s., M*_panic words_ = 0.1, *M*_neutral words_ = 0.1, controls.

Overall delta-eVAS values were rather low and following Clark‘s definition of a panic attack as “the sudden increase in anxiety reaching at least 50 on a 100 point scale” (p155) [7], we can conclude that none of the patients had a panic attack.

Correlations between MADRS scores and mean differences scores between panic and neutral word pairs within the patient groups were not significant different, *r_s = _*0.24, N = 20, *n.s.*; *r*_s = _0.13, N = 20, *n.s.* Further, after excluding the panic patients with a co-morbid depressive disorder the frequency of panic attacks is still zero (*M*_panic_ = 2, N = 11). No significant differences between panic and neutral word associates were found (*z* = .98, N-Ties = 8, *p*>.32). Also no differences between the patient groups were found, U = 75, N_1_ = 11, N_2_ = 15, *p*>.7.

## Discussion

To enhance the ecological validity of our study we first conducted a stimulus validation experiment. The results of this led to the selection of a number word pairs that appeared to induce anxiety. Next, we presented these anxiety inducing word pairs repeatedly to panic disorder patients, under the hypothesis that they would trigger panic attacks. Indeed, PD patients were more anxious when reading these word pairs, compared to neutral word pairs. Furthermore, regardless of the presentation of word associates patients were also more anxious and reported more symptoms than control participants. Both these observations confirm the ecological validity of the experimental setup. However, none of the participants experienced a panic attack upon reading the word pairs. Second, when comparing the anxiety response between PD patients and participants with other anxiety disorders, no significant differences were found. These findings are in contrast with a previous report in which 83% of the PD patients were reported to have a panic attack after reading similar word pairs [4].

To explain this discrepancy the following issues may be taken into account: (1) severity of PD, (2) interference of co-morbid depressive complaints, (3) sample size and (4) physical symptom reports. An accurate comparison is difficult however, since the previous report does not contain complete information in this regard. First, in the present study, panic diagnosis and severity was assessed using standardized scales as well as clinical diagnosis by structured interview. All PD patients had at least moderate to severe panic disorder, in clear contrast with the ‘anxious control’ group. Further, there were no differences in experimental anxiety, trait anxiety and depressive symptoms between the patients, indicating that the panic patients had substantially higher levels of symptomatology related to panic and agoraphobia. The overall disorder severity of both patient groups is comparable based on the scores on the STAI-T. Moreover the panic patients in the present study seem also very comparable with former panic studies. In a recent study by Kircher et al. [40], the PD patients’ scores on the PAS were similar to the present study: 25.97 (23.27–28.68) vs. 25.5 (*SD* = 10.3). Other studies concerning PD used the Panic Disorder Severity Scale [41] and STAI to assess disorder severity. On the basis of the STAI, the present panic group seems very comparable with former PD studies. Present study: 51 (10.7) vs. 51. 8 (12.1) Kroeze & van den Hout [42] vs. 39.89 (10.80) Lissek et al. [43]. Further in provocation studies with for instance the 35% CO_2_ panic challenge in PD severity of illness is also comparable based on the STAI-T: 47.4 (13.6) = smokers/44 (11.6) = non-smokers [44]. It is therefore unlikely that insufficient panic severity can explain the lack of panic induction in the present study. Second, it is conceivable that depressive symptoms, like a lowered mood state and concentration deficits, could have influenced the panic reaction in the present experiment. In the research of attentional bias, suppression of the stroop effect was found in social phobia with co-morbid depression [45], [46] as well as in depressive patients without co-morbid anxiety disorder [47]. However, no significant correlations were found between depressive symptoms and anxiety scores. Moreover, no differences could be demonstrated regarding number of panic attacks or level of anxiety when panic patients with co-morbid depression were excluded from the analyses. Panic disorder severity nor depression scores were mentioned in the previous report [4]. Third, theoretically, it is possible that our sample size was insufficient. However, only 12 PD patients, 8 recovered PD patients and 12 healthy controls participated in this previous experiment, compared to 20 PD patients, 20 anxious controls and 30 healthy controls in the present study. Other experimental studies with similar sample size were able in demonstrating their conditioning or generalization effect in healthy volunteers [48], [49] and in PD [43] patients. Furthermore, a sample size of 8 participants per group has 90% power to detect an effect such as reported by Clark (with α = 0.05). Which implies that our sample was large enough to demonstrate an effect. Fourth, the present study was developed to test the causality hypothesis: catastrophic cognitions of physical sensations are regarded as sufficient and necessary in the production of panic attacks. In this regard catastrophic misinterpretations can only occur in the presence of bodily sensations. One can argue if in the present study these sensations were indeed present. However, following the cognitive theory, where is stated that only the presence of certain physical sensations is necessary, regardless of their origin, it was to be expected that in the present experiment certain physical sensations would be present. This was confirmed by the finding that symptom scores were elevated at the start and at the end of the experiment. Moreover, in the present study we presented these particular word associates in participants which already had a learning history of panic attacks, anxiety and avoidance behavior. Thus the presented word-associates were not neutral, but already fear-relevant and associated in memory. Following Lang [50], the presentation of the word associates will therefore immediately activate the fear network whereby the related network of fear-responses (hypertension, hyperventilation …) will also be activated. Furthermore, one could argue that the actual presence of bodily sensations is necessary to test the causality hypothesis. However, following cognitive theory, the presence of a bodily symptom is indeed necessary at the start of the disorder. One has to really feel a bodily sensation, which will be interpreted in a catastrophic fashion, which will further lead to anxiety and this leads to further symptoms and so on, spiraling into a vicious circle. However when the vicious circle is already present, symptoms are not necessary any more. For instance, interpretations can then be enough to activate tension or anxiety. After a period of time sensations are not necessary anymore to activate this circle. Then it is even sufficient to think about panic, to evoke symptoms. Our PD group had already a learning history of panic attacks, anxiety and avoidance, meaning that in this group relevant word pairs were sufficient to evoke bodily symptoms.

Some limitations of the present study need to be taken into account. First, it is important to acknowledge that one of the limitations of the cognitive model of panic is that it can be difficult to test. For instance, Roth, Willhelm and Petit [28] noted that catastrophic cognition theories can seem unfalsifiable because of the abstract nature of concepts such as catastrophic misinterpretations, which are challenging to effectively measure. Further, given that panic involves multiple response systems [51] relying on only self-report is a limitation of the present study. Moreover, since in cognitive theory is stated that the misinterpretation of physical sensation is central, the absence of a continuous assessment of these physical symptoms, for instance through psychophysiology measurements and self-report is a clear limitation.

In sum, despite the use of carefully selected anxiety inducing word pairs, appropriate sample size, presence of an anxious control group and controlling for depressive symptoms, we were not able to find support for the ‘causality hypothesis’ in cognitive theory to explain the role of catastrophic cognitions in the occurrence of panic attacks. However, this does not mean that catastrophic thinking does not play a role in the broader concept of panic disorder, for this condition is characterized by anticipatory anxiety and avoidance behavior besides panic attacks. It is very likely that this kind of thinking plays a central role in the anticipatory anxiety that characterizes panic disorder patients. On the basis of phenomenological and neurobiological research Bouton et al [26] make a clear distinction between panic and anxiety as two aversive motivational states. It is suggested that panic attacks are descriptively and functionally distinct events when compared with anxiety. Panic refers to a subjective sense of extreme fear, which is accompanied by a massive autonomic surge and strong fight or flight response. In contrast, anxiety refers to apprehensive anticipation of future danger (the danger is not ‘actually’ present), accompanied by somatic symptoms of tension, vigilance, and worrying. Following this distinction catastrophic misinterpretations seem more related to anxiety than to panic. Catastrophic thinking (and worrying) mostly refers to the anticipation of physical sensation, such as an acute medical condition with fatal result. PD patients were indeed more anxious upon reading panic word pairs (compared to neutral word pairs), but were not in a state of acute panic. Further, the ‘anxious control’ group was equally anxious than the PD patients, indicating that catastrophic thinking seems capable of provoking anxiety in most anxiety disorders. Therefore it seems reasonable to assume that catastrophic thinking plays an important role in most anxiety disorders, in line with cognitive theory of anxiety disorders. It is also the basis of some successful interventions for those disorders.

In conclusion, on the basis of our results it appears unlikely that catastrophic thinking has a central role in the immediate development of panic attacks. Anxiety can however contribute to the evolution of sporadic panic attacks towards PD through mechanisms such as interoceptive conditioning [26], [52], [53]. For future research it would be interesting to include psychophysiology measurements besides the use of self-report to reveal a more comprehensive picture of the PD pathology. Furthermore, it would be interesting to focus more on the anxiety processes induced by catastrophic thinking or interoceptive conditioning [54] in the development into PD. In the learning theory of Bouton, Mineka and Barlow [26] a prominent role is given to interoceptive fear conditioning to explain the evolution of a panic attack into a PD. Through interoceptive conditioning bodily sensations can act as a predictor for a panic attack and evoking conditioned anxiety. Specifically, the initial interoceptive precursors of a panic attack (such as sweating, palpitations…) become conditioned stimuli, predicting more intense arousal and provoking anxiety as a conditioned response. This anxiety response may produce additional and more intense interoceptive stimuli (more palpitations, sweating, faster breathing) that further trigger and potentiate anxiety, spiraling into panic. Anxiety thus becomes a precursor of panic.
